# Apolipoprotein A-I priming via SR-BI and ABCA1 receptor binding upregulates mitochondrial metabolism to promote insulin secretion in INS-1E cells

**DOI:** 10.1371/journal.pone.0311039

**Published:** 2024-11-15

**Authors:** Claire L. Lyons, Elaine Cowan, Oktawia Nilsson, Manca Mohar, Pablo Peña-Martínez, Lena Eliasson, Jens O. Lagerstedt

**Affiliations:** 1 Department of Experimental Medical Sciences, Unit of Medical Protein Science, Lund University, Lund, Sweden; 2 Department of Clinical Sciences, Unit of Islet Cell Exocytosis, Lund University Diabetes Centre, Malmö, Sweden; 3 Division of Clinical Genetics, Department of Laboratory Medicine, Lund University, Lund, Sweden; Faculty of Pharmacy, Ain Shams University, EGYPT

## Abstract

Apolipoprotein A-I (ApoA-I), the primary component of high-density lipoprotein (HDL) cholesterol primes β-cells to increase insulin secretion, however, the mechanisms involved are not fully defined. Here, we aimed to confirm ApoA-I receptors in β-cells and delineate ApoA-I-receptor pathways in β-cell insulin output. An LRC-TriCEPS experiment was performed using the INS-1E rat β-cell model and ApoA-I for unbiased identification of ApoA-I receptors. Identified targets, alongside ATP binding cassette transporter A1 (ABCA1) (included control) were silenced in the same cells, and insulin secretion (ELISA) and mitochondrial metabolism (seahorse) were assessed with/without ApoA-I priming. Human β-cell expression data was used to investigate ApoA-I receptor pathways in type 2 diabetes (T2D). Scavenger receptor B1 (SR-BI) and regulator of microtubule dynamics 1 were identified as ApoA-I targets. SR-BI or ABCA1 silencing abolished ApoA-I induced increases in insulin secretion. ApoA-I priming increased mitochondrial OXPHOS, however this was greatly attenuated with SR-BI or ABCA1 silencing. Supporting this, human β-cell expression data investigations found SR-BI and ABCA1 to be correlated with genes associated with mitochondrial pathways. In all, SR-BI and ABCA1 correlated with 73 and 3 genes differentially expressed in T2D, respectively. We confirm that SR-BI and ABCA1 are the primary β-cell ApoA-I receptors and demonstrate that ApoA-I priming enhances β-cell insulin secretion via the upregulation of mitochondrial metabolism through ApoA-I-SR-BI and ApoA-I-ABCA1 pathways. We propose that SR-BI relies on mitochondrial and exocytotic pathways, while ABCA1 depends solely on mitochondrial pathways. Our findings uncover new targets in ApoA-I β-cell mechanism for T2D therapies.

## Introduction

Limited insulin release from pancreatic β-cells underpins type 2 diabetes pathology [1] and the global epidemic of diabetes is expected to affect 643 million by 2030 [2]. Apolipoprotein A-I (ApoA-I), the primary component of high-density lipoprotein (HDL) is best known for its role in cholesterol and lipid uptake, and transport in the circulation [3]. Both HDL and ApoA-I have also been implicated in glucose control. Clinical studies report low HDL and ApoA-I levels increase type 2 diabetes risk [4, 5], and infusion of HDL into people with type 2 diabetes has been found to decrease plasma glucose while simultaneously increasing plasma insulin [6]. Furthermore, several functional studies show ApoA-I positively affects glucose homeostasis and metabolic health via increasing skeletal muscle glucose uptake and pancreatic β-cell insulin secretion [7–13]. For instance, our group demonstrated ApoA-I improves glucose tolerance by equally increasing insulin secretion and glucose clearance in mice [7, 9, 11], and stimulates glucose uptake in skeletal and cardiac muscle [7, 10]. We also confirmed that truncated ApoA-I peptide RG54 retained ApoA-I biological activity with respect to enhanced glucose control in insulin resistant and diabetic mice, insulin secretion from INS-IE cells and glucose uptake in human myotubes [14]. Additionally, others convincingly report ApoA-I mediated increases in glucose uptake in skeletal muscle cells are dependent on ATP binding cassette transporter A1 (ABCA1) and scavenger receptor B1 (SR-BI) transmembrane receptors [12].While progress has been made in delineating ApoA-I pathways in insulin secretion, the exact mechanisms are still undefined. Our more recent investigations with clonal INS-1E cells and mouse islets found ApoA-I efficiently primes β-cells to increase glucose stimulated insulin secretion (GSIS). We showed that this priming step increases pancreatic and duodenal homeobox 1 (PDX1) shuttling to the nucleus, pro-insulin processing and insulin granule accumulation at the plasma membrane. Hence, we demonstrate that ApoA-I exerts at least some of its effects on GSIS by influencing β-cell insulin biosynthesis and exocytosis [8].

Here we aimed to substantially build on the findings of our previous investigations to identify the primary receptors for ApoA-I in β-cells and fully delineate the mechanisms of ApoA-I priming leading to enhanced insulin secretion. Firstly, we performed a TriCEPS^™^-based ligand-receptor capture (LRC-TriCEPS) experiment in INS-1E β-cells to identify putative ApoA-I receptors. Thereafter we evaluated insulin secretion and mitochondrial oxidative phosphorylation (OXPHOS) in ApoA-I receptor knockout and wildtype INS-1E cells primed with and without ApoA-I to validate identified ApoA-I targets and investigate if ApoA-I increases β-cell GSIS by upregulating mitochondrial metabolism. Finally, to relate our findings to human physiology, we exploited publicly available β-cell expression data from human donors to define ApoA-I’s underlying pathways.

## Materials and methods

### INS-1E culture

INS-1E cells, a pancreatic β-cell model of rat insulinoma origin [15] (gifted by Prof. Claes Wollheim, University of Geneva) were cultured in RPMI Glutamax (Gibco) supplemented with 1% penicillin/streptomycin (Invitrogen), 10% heat inactivated FBS (HyClone), and 50 μM β-mercaptoethanol (Invitrogen) at 37 ºC in a 5% CO_2_ humidified atmosphere.

### ApoA-I purification

Human ApoA-I with N-terminal His-tag was expressed in *E*. *coli* and purified as previously described [16, 17] (S1 Fig in File). ApoA-I was either produced in-house or by Lund University Protein Production Platform (Sweden).

### Circular dichroism, dot blot and flow cytometry pretest of ligand-receptor glycocapture (LRC-TriCEPS^™^)

#### Circular dichroism

ApoA-I prepared in 25 mM HEPES pH 8.2 and PBS pH 7.4 was incubated with glycine at 23 ºC for 90 min and then again for 20–40 min at 350 rpm in a Thermomixer (Eppendorf 5350). Incubated and non-incubated samples (0.5 mg/ml) were analysed on a spectropolarimeter (Jasco J-810) with a Peltier set to 25 ºC (Jasco CDF-426S) to determine secondary structure. Spectra were measured at 200–260 nm in 1 nm wavelength increments and were corrected by removing the signal of the background buffer.

#### Ligand coupling for dot blot

20 μg transferrin (positive control) and glycine (negative control) in 25 mM HEPES pH 8.2, and ApoA-I in PBS pH 7.4 were incubated with biotinylated TriCEPS^™^ (TriCEPS v2.0) for 90 min at 350 rpm in a thermomixer. Samples were then incubated for a further 20 min (transferrin and glycine) or 40 min (ApoA-I) before adding to a nitrocellulose membrane at 1:10, 1:100 and 1:1000 (LC2000, Invitrogen). The membrane was dried, blocked in 5% BSA in TBS-T for 30 min, and incubated with streptavidin HRP (Thermo Fisher Scientific) (1:66 000) for 1 h in the dark. Finally, it was washed 3 times in TBS-T, incubated with SuperSignal^™^ West Femto Maximum Sensitivity Substrate (Pierce) and developed with the Licor system.

#### Ligand coupling for flow cytometry

INS-1E cells seeded at 1x10^6^ cells/ml and cultured for 48 h were washed with PBS and incubated with the TriCEPS (v2) coupled-ligands (transferrin, glycine and ApoA-I at 20 μg) under 3 conditions (22 ºC for 30 min in PBS pH 7.4; 4 ºC for 90 min in PBS pH 7.4 or 4 ºC for 90 min in PBS pH 6.5) at 350 rpm in a thermomixer. After washing with PBS, they were incubated with Alexa Fluor 647 Streptavidin (Thermo Fisher Scientific) (1:100) for 30 mins at 4 ºC in the dark. Cells were then washed, collected, centrifuged (2000 g for 3 mins) and washed again. They were finally strained, centrifuged (3000 g for 5 min) and re-suspended in PBS before analysis by flow cytometry (LSRFortessa, BD Biosciences).

### LRC-TriCEPS^™^

INS-1E cells were seeded at 15x10^6^ cell/15 cm plate (1 plate/treatment arm). Transferrin, glycine, and ApoA-I (300 μg) were coupled to TriCEPS^™^ (TriCEPS v3.0) in PBS pH 7.4 and incubated for 90 min at 350 rpm. 24 h after seeding, cells were washed with PBS pH 7.4 and oxidised with 75 mM sodium metaperiodate in ice cold PBS pH 6.5 for 15 min at 4 ºC in the dark under gentle agitation. Cells were then washed with PBS pH 7.4, incubated with the TriCEPS-coupled samples for 90 min at 4 ºC in the dark under gentle agitation, detached using EDTA, centrifuged at 300 g for 5 min at 4 ºC and resuspended in ice cold LRC buffer. They were finally separated into triplicates (triplicates/treatment arm (n = 3 biological replicates)) and centrifuged at 300 g for 5 min at 4 ºC before supernatants were collected and stored at -80 ºC.

The following were conducted by DualSystems Biotech AG (Switzerland): Cells were lysed, and proteins were purified using solid-phase chromatography, washed and trypsinised before LC-MS/MS (Thermo Orbitrap Elite spectrometer with electrospray ion source) was performed in data dependent acquisition mode (TOP20) using a 15 cm C18 packed column and an 80 min gradient. Progenesis software was applied for alignment and feature detection and the Comet search engine for spectra identification. The annotated Rattus norvegicus database (Uniprot) and the protein sequences for human ApoA-I and transferrin were used for analysis. Results were filtered for membrane associated proteins. See 2.11 for statistics.

### siRNA transfection

A *Silencer*^*®*^ Select siRNA (siRNA) targeting SR-BI *(siScarb1)*, ABCA1 *(siAbca1) or RMD1 (siRmd1)* (Thermo Fisher Scientific) and a non-targeting siRNA *(siNeg)* were used at a final concentration of 10 nM. INS-1E cells were seeded at 200 000 cells/well in antibiotic free media. 24 h later, fresh antibiotic free media was added with Lipofectamine RNAiMAX (Thermo Fisher Scientific) with the relevant siRNA in optiMEM media and cells were incubated until further investigation.

### cDNA and RT-PCR

48 h post transfection RNA was isolated (n = 5 biological replicates /group) using the RNeasy kit (Qiagen), and cDNA synthesis was performed with 500 ng template RNA using high-capacity cDNA synthesis kit (Thermo Fisher Scientific) according to manufacturer instructions. Taqman probes (housekeeping probes: *Ppia* Rn00690933_m1 (PPIA) and *Hprt1* Rn01527840_m1 (HPRT1); target probes: *Scarb1* Rn00580588_m1 (SR-BI), *Abca1* Rn00710172_m1 (ABCA1) and *Rmd1* Rn01440098_m1 (RMD1); Thermo Fisher Scientific), 100 ng cDNA and water were prepared, and RT-PCR was conducted (7500 Real Time PCR System, Applied Biosystems) as per manufacturer protocol.

### Western immunoblotting

Total protein was extracted for control *(siNeg)* and SR-BI *(siScarb1)* or ABCA1 *(siAbca1)* silenced cells 72 h post transfection (n = 4 biological replicates/group): cells were washed in ice-cold PBS and lysed with RIPA buffer (1% NP-40, 50 mM Tris-HCl pH 7.4, 0.25% Na-deoxycholate, 1 mM EDTA and 150 mM NaCl) supplemented with protease inhibitor cocktail (Sigma) for 20 min. Lysates were centrifuged (4700 g for 30 min at 4 ºC) and protein content determined by BCA assay (Thermo Fisher Scientific) as per manufacturer instructions.

Thereafter, separation (20 μg/sample) was performed by SDS-PAGE on 4–20% kDa Mini-PROTEAN^®^ TGX Precast Gels Any kDa (Bio-Rad). The gels were activated with ultraviolet light for 1 min to visualize total protein on blotted membranes after transfer (Bio-Rad). Proteins were transferred onto polyvinylidene difluoride membranes using the TransBlot^®^ Turbo^™^ Transfer System (Bio-Rad). After blocking for 1 h at room temperature in 1xTBS and 5% (wt/vol) non-fat dry milk, membranes were incubated overnight at 4 °C with primary antibodies (Anti-SR-BI antibody, ab217318, 1:2000, Abcam (Blot 1: *Scarb1* silenced samples)) and (Anti-ABCA1 antibody, #96292, 1:1000, Cell Signaling Technology (Blot 2: *Abca1* silenced samples)) in 1 x TBS, 0.05% (vol/vol) Tween 20 and 1% (Blot 1) or 5% (Blot 2) (wt/vol) non-fat dry milk. Membranes were then washed and the antigen—antibody complex(s) detected by incubating for 1 h at room temperature with horseradish peroxidase (HRP)-conjugated anti-rabbit IgG secondary antibody (170–6515, 1:3000, Bio-Rad). Clarity Western ECL Substrate was used to visualize antibody binding with a ChemiDoc XRS+ System (Bio-Rad). The signal intensity of each protein band was measured with Image Lab software (version 5.2.1; Bio-Rad) and normalized to that of the total protein bands in the lane.

### Glucose-stimulated insulin secretion (GSIS)

INS-1E cells (200 000/well) were washed in secretion assay buffer (SAB; 120 mM NaCl, 5 mM KCl, 2.5 mM CaCl_2_, 1.2 mM KH_2_PO_4_, 1.2 mM MgSO_4_, 25 mM NaHCO_3_, 10 mM HEPES and 0.1% BSA) containing 3.3 mM glucose, 72h post transfection (n≥4 biological replicates/group). They were then incubated with fresh SAB containing 3.3 mM glucose with or without 0.5 mg/ml ApoA-I for 2 h (ApoA-I priming). This was exchanged for SAB containing 3.3- or 20-mM glucose and incubated again for 1h. Supernatants were centrifuged (2000 g for 10 min) and insulin measured by ELISA (Mercodia) as per manufacturer’s instructions and protein content was determined in collected lysates as in Section 2.7.

### Mitochondrial respiration (OXPHOS)

INS-1E cells (120 000/well) were seeded on seahorse plates (Agilent) and either left to remain in culture or transfected after 24 h (n = 5 technical and n≥4 biological replicates/group). 96 h post seeding, cells were primed with ApoA-I (none, 0.1, 0.5 or 1 mg/ml) in assay buffer (114 mM NaCl, 4.7 mM KCl, 1.2 mM KH_2_PO_4_, 1.16 mM MgSO_4_, 20 mM HEPES and 2.5 mM CaCl_2_, pH 7.2) with 3.3 mM glucose for 2 h at 37 °C in a CO_2_ free incubator. The seahorse calibration plate (Agilent) was similarly incubated with calibration solution (Agilent) for 12–24 h. After priming, the buffer was exchanged to fresh assay buffer with 3.3 mM glucose. To determine changes in mitochondrial respiratory response/OXPHOS, oxygen consumption rate (OCR) for each well was immediately measured by the XFe24 Extracellular Flux Analyzer (Agilent) at basal 3.3 mM glucose and, after sequential addition of 20 mM glucose, 4 μg/ml oligomycin, 2 μM FCCP and 1 μM rotenone respectively, as previously described [18]. Protein content was determined in collected lysates as in Section 2.7. Primary data analysis was performed using the Seahorse Analytics webtool (https://seahorseanalytics.agilent.com/).

### SR-BI and ABCA1 pathway investigations in human β-cell data

β-cell gene expression data was extracted from a pancreatic islet single cell sequencing dataset (GSE153855 accessed 05/04/2023) from which we could not identify individual participants [19], and a variance stabilizing transformation applied using Deseq2 [20]. As the data used was publicly available and we were not involved in the study data collection we did not seek approval from any ethics committee/review board. Differential analysis was performed with data from donors with (n = 6: n = 3 males, n = 3 females) vs without type 2 diabetes (n = 5: n = 3 males, n = 2 females) and SR-BI and ABCA1 expression were correlated with all differentially expressed genes. Pearson’s correlation and a threshold FDR of below 10% (q<0.1) by Benjamini-Hochberg correction was applied. All analyses were performed in RStudio (Version 4.2.2).

MitoCarta3.0 (https://www.broadinstitute.org/mitocarta/mitocarta30-inventory-mammalian-mitochondrial-proteins-and-pathways) and WebGestalt (https://www.webgestalt.org/) were used to investigate pathways associated with SR-BI and ABCA1 expression changes on the basis of significantly correlated gene lists. All significantly correlated genes were searched in MitoCart3.0 to assign annotated mitochondrial pathways. Gene set enrichment analysis (GSEA) was performed with all SR-BI and ABCA1 significantly correlated genes in WebGestalt against Gene Ontology Molecular function databases with enrichment thresholds set at a minimum of 3 genes and an FDR below 5% (q<0.05).

### Statistics

DualSystems Biotech used the Trans proteomic pipeline to validate putative identifications and protein inference in the LRC-TriCEPS experiment. Ion extracted intensity was used for relative quantification and an ANOVA with multiple testing to determine differential protein abundance. Adjusted p-values were plotted against the magnitude of fold enrichment values.

For all other generated data, values are expressed as mean ± SD unless otherwise stated. GraphPad Prism (Version 9.3.1) was used for statistical analysis where paired t-test or paired or unpaired one-way ANOVA with Dunnett’s or Bonferroni’s posthoc testing was applied and p<0.05 was considered significant. In addition, data were also assessed for outliers by the ROUT testing method (Q = 1%).

## Results

### Potential targets/receptors for ApoA-I are identified in INS-1E cells

**Fig 1:**
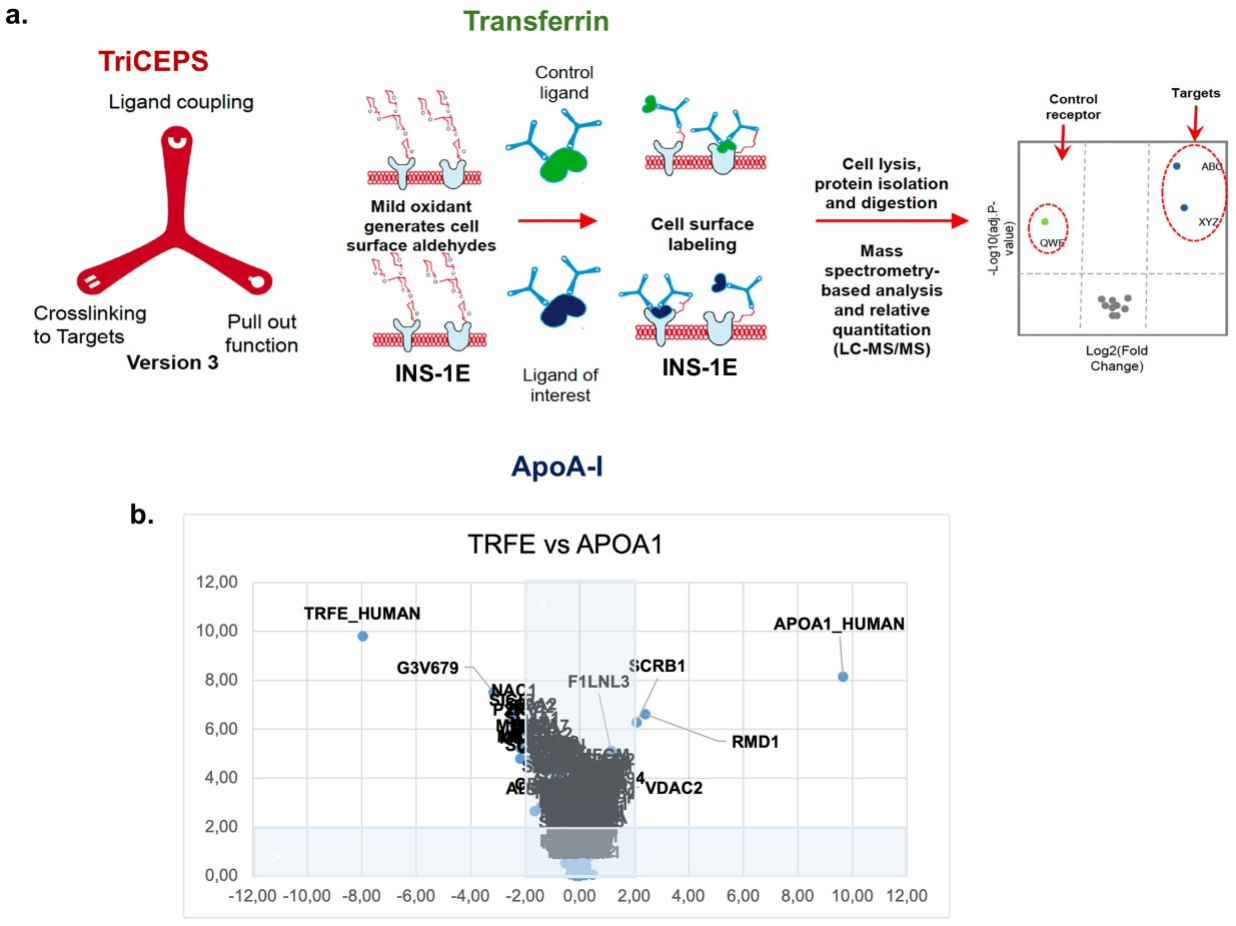
Identification of potential ligands for ApoA-I. a. Schematic of the experimental set up and design of the TriCEPS experiment in INS-1E cells. b. Proteins enriched in the positive control transferrin and the ligand of interest, ApoA-I, are displayed in a volcano plot whereby the x-axis is the mean ratio fold change (log2 scale), and the y-axis represents the statistical adjusted p-value for the ratio fold change (-log10 scale); ANOVA model with adjustment for multiple pairwise comparisons, n = 3 biological replicates for each sample.

To identify ApoA-I targets, cross-linked proteins in INS-1E were analysed by LC-MS/MS (Fig 1a). Firstly, a trifunctional chemoproteomics reagent (TriCEPS^™^, [21, 22]) which binds to the glycans of both a ligand and its targets was coupled to ApoA-I. Preliminary experiments determined the optimal buffer, confirmed successful TriCEPS binding to the ligands of interest and demonstrated the optimal incubation settings (S2 Fig in S1 File). In total, 399 membrane associated proteins were identified of which 264 contained more than 2 peptides and were used for statistical analysis. Successful targets had a minimum of 2 identified peptides (log2 fold change (FC) >2 and adjusted p-value of <0.01). Transferrin (TRFE) and its receptor (G3V679) were enriched in the positive control. For ApoA-I, scavenger receptor class B member 1 (SR-BI *Scarb1*; log2 FC 2.1; adj p-value 5.3E-07) and regulator of microtubule dynamics protein 1 (RMD1 *Rmdn1*; log2 FC 2.4; adj p-value 2.4E-07) were identified (Fig 1b and Table 1) as targets. As expected ApoA-I Human was present in the ApoA-I sample. In addition, F1LNL3, the rat ortholog for ATP-binding cassette 1 (ABCA1 *Abca1*; log2 FC 1.14; adj p-value 7.7E-06) was also included as a control target in further experiments given it has a known role in ApoA-I action [23]. Peptide expression profiles showing the relative abundance of identified peptides in the transferrin and ApoA-I samples are presented in Fig 1b. Proteins were imported on Protter [24] to generate protein topology plots for ApoA-I identified targets (S3 Fig in S1 File).

**Table 1:** Control and target proteins identified for transferrin and ApoA-I in INS-1E cells through ligand receptor coupling.

Enriched in	Protein name	Accession	Gene Name	Protein description	log 2 (FC)	adj p-value	-log10 (adj p-value)
**Transferrin**	TRFE_ HUMAN	P02787	TF	Serotransferrin	-8.0	1.6E-10	9.8
**ApoA-I**	SCRB1	P97943	Scarb1	Scavenger receptor class B member 1	2.1	5.3E-07	6.3
	RMD1	Q4G069	Rmdn1	Regulator of microtubule dynamics protein 1	2.4	2.4E-07	6.6
	APOA1_HUMAN	P02647	APOA1	Apolipoprotein A-I	9.7	7.1E-09	8.2
	F1LNL3	F1LNL3	Abca1	ATP-binding cassette subfamily A member 1	1.14	7.7E-06	5.11

### Silencing of SR-BI and ABCA1 in INS-1E cells confirm their roles in increasing GSIS upon ApoA-I priming

**Fig 2:**
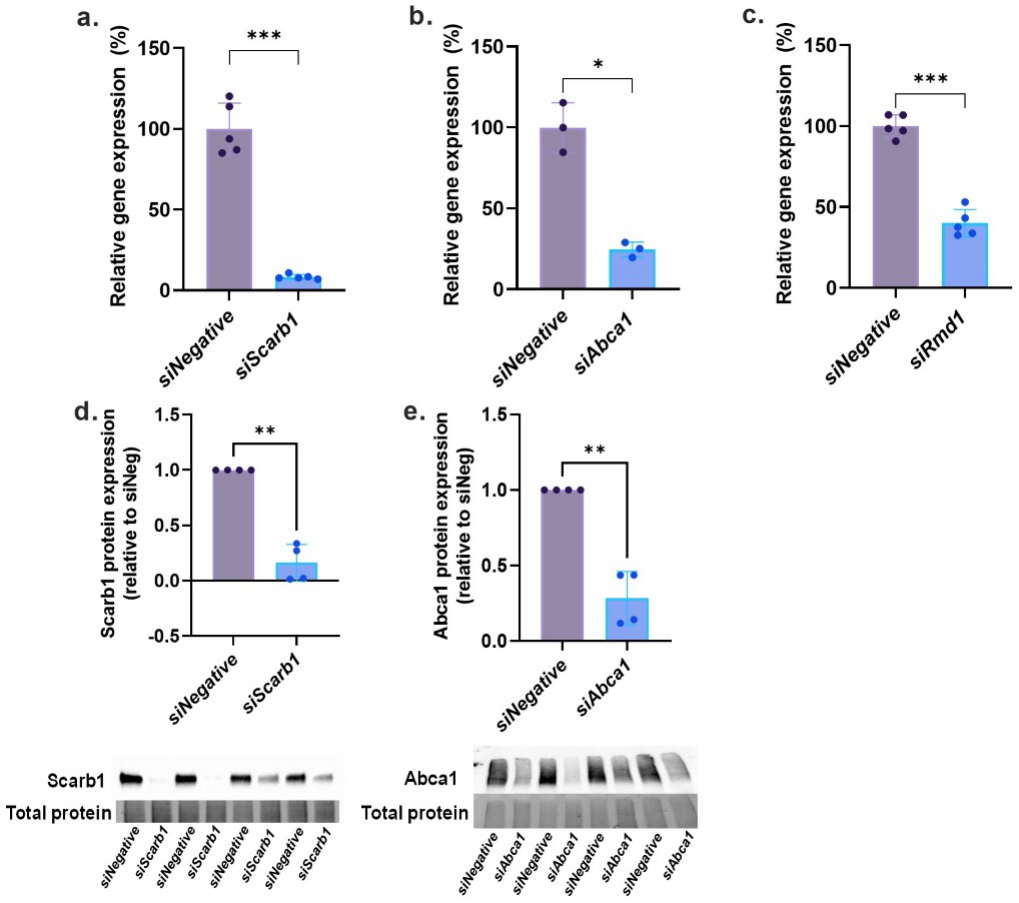
Confirmation of silencing of target receptors. Gene expression analysis of a. *siScarb1* (SR-BI), b. *siAbca1* (ABCA1) and c. *siRmd1* (RMD1) (n = 5) silencing in INS-1E cells. Protein expression analysis and representative bands of d. *siScarb1* (SR-BI) and e. *siAbca1* (ABCA1) silencing in INS-1E cells (n = 4 for each). All values represent mean ± SD; *p<0.05, **p<0.01 and ***p<0.001; paired t-test.

Potential targets for ApoA-I were silenced using silencing RNA (Fig 2). SR-BI was reduced at both the mRNA (Fig 2a) and protein level (Fig 2d). Similarly, ABCA1 was reduced at both the mRNA (Fig 2b) and protein level (Fig 2e). Despite successful knockdown of RMD1 mRNA (Fig 2c), this target was undetectable at the protein level in both *siNeg* and knockdown samples. Western immunoblots are shown in S4 Fig in S1 File.

**Fig 3:**
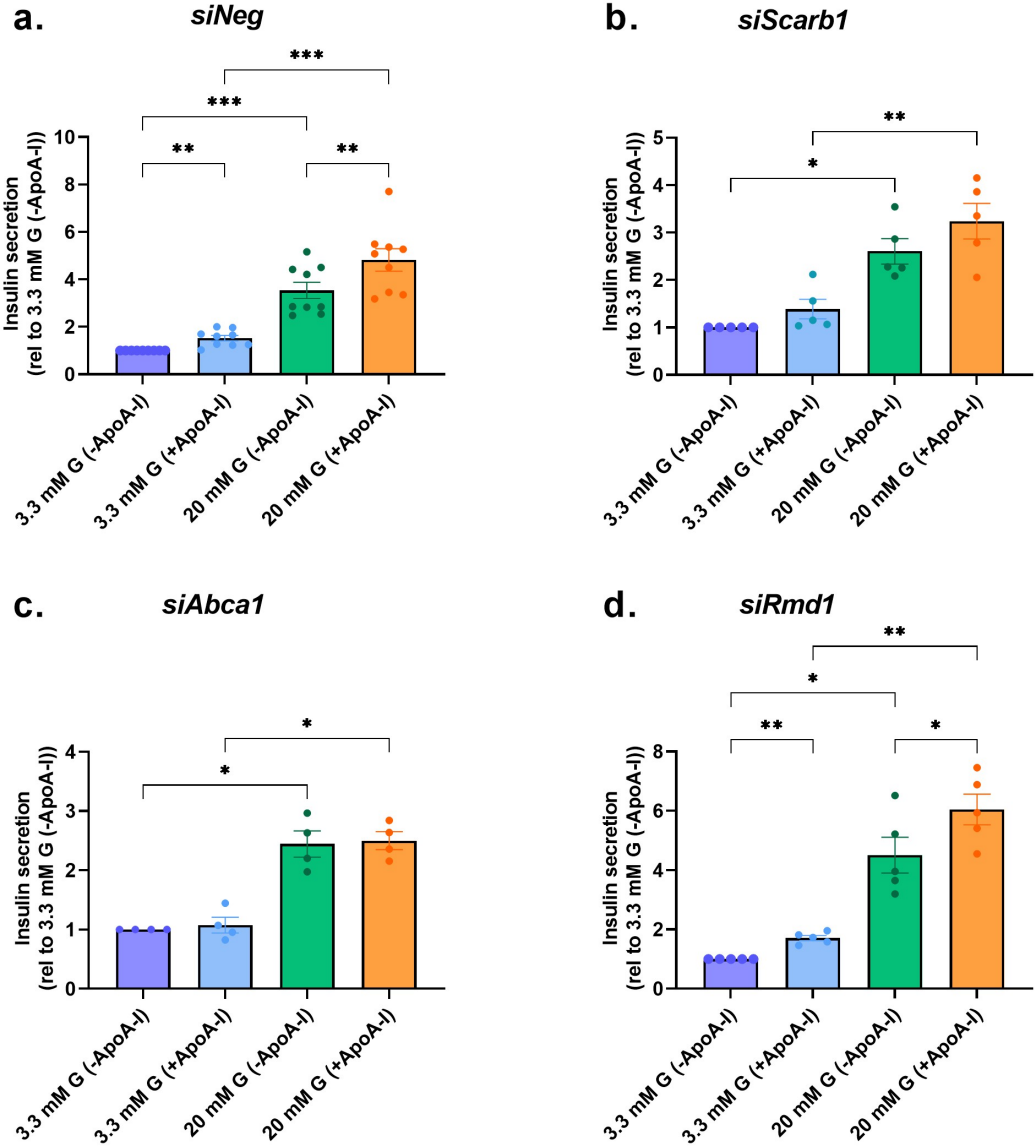
Glucose stimulated insulin secretion (GSIS) in INS1-E KD cells primed ± ApoA-I. GSIS assessment in a. *siNeg* (Control) (n = 9), b. *siScarb1* (SR-BI) (n = 5), c. *siAbca1* (ABCA1) (n = 4) and d. *siRmd1* (RMD1) (n = 5) silenced INS-1E cells primed with or without ApoA-I. All values represent mean ± SD; *p <0.05, **p<0.01 and ***p<0.001; paired one-way ANOVA with Bonferroni posthoc test.

The effect of silencing on insulin secretion was investigated. The insulin secretory response was increased with increased concentration of glucose (Fig 3) and inclusion of ApoA-I priming at 20 mM further potentiated this in the *siNeg* control by 28% (Fig 3a). When SR-BI or ABCA1 is silenced, GSIS under 20 mM glucose with ApoA-I priming is no longer significantly increased indicating SR-BI and ABCA1, are involved in mediating the effect of ApoA-I (Fig 3b and 3c). However, when RMD1 is silenced, ApoA-I still significantly increases insulin secretion (Fig 3d). The role of RMD1 in other ApoA-I functions was not further analysed.

### ApoA-I priming increases mitochondrial OXPHOS in INS-1E cells

**Fig 4:**
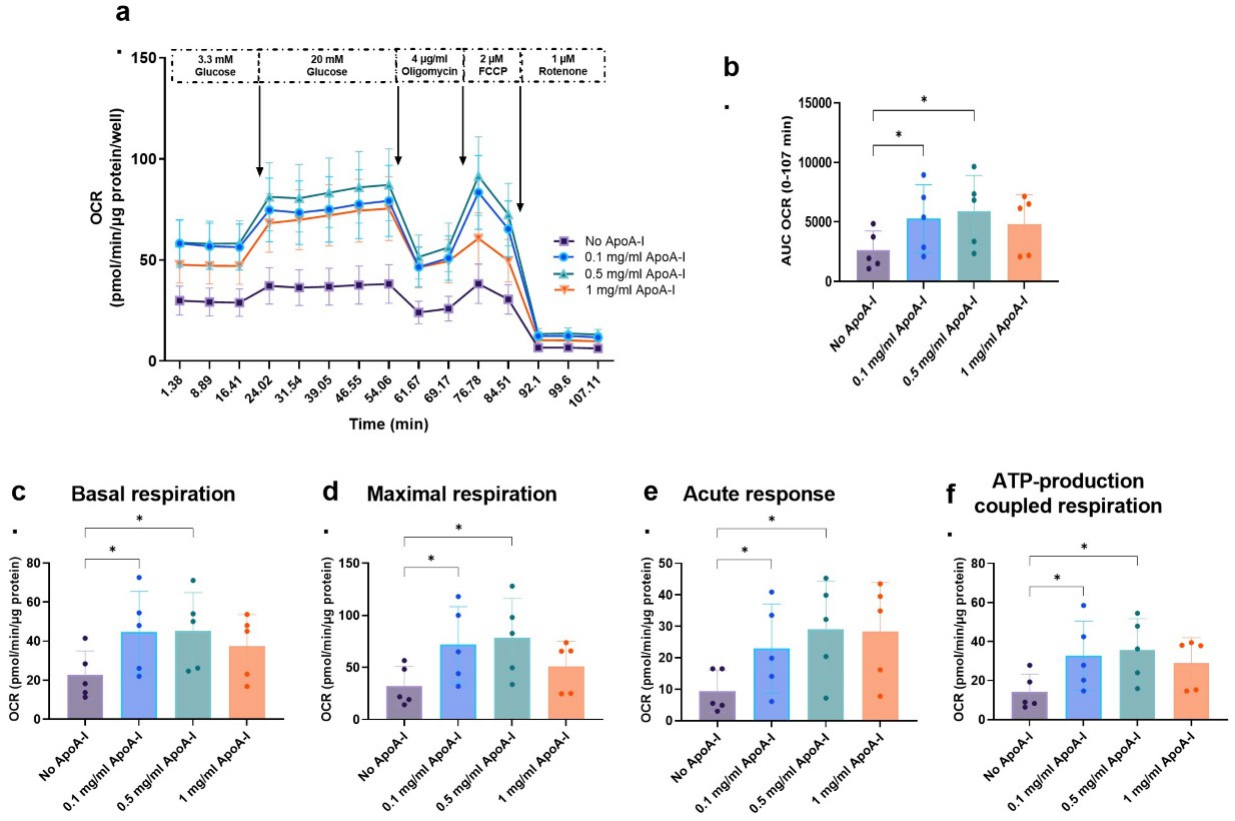
Effects of ApoA1 priming on oxidative phosphorylation (OXPHOS) in cultured INS-1E cells. a. OCR (oxygen consumption rate) and b. AUC for OCR, c. basal respiration, d. maximal respiration, e. acute response and f. ATP-production coupled respiration in INS-1E cells with or without ApoA-I priming (0.1mg/ml, 0.5mg/ml or 1mg/ml) for 2h (n = 5). Each value represents mean ± SD, except in a where it is mean ± SEM; *p<0.05; paired one-way ANOVA with Dunnett’s posthoc test.

Due to the critical role the mitochondria play in insulin secretion [1], we used a Seahorse XFe24 Analyser to determine if ApoA-I mediates its effect on GSIS via effects on mitochondrial metabolism (Fig 4 and S5 Fig in S1 File). Mitochondrial OXPHOS as measured by OCR was clearly positively impacted by ApoA-I priming (Fig 4a) ApoA-I priming increased OCR at 0.1 and 0.5 mg/ml ApoA-I (Fig 4b). Increases in basal respiration, maximal respiration, acute response, and ATP-coupled respiration (Fig 4c–4f) were also observed in cells primed with ApoA-I at 0.1 mg/ml and 0.5 mg/ml compared to no ApoA-I.

### SR-BI and ABCA1 silencing reduced the increased mitochondrial OXPHOS observed after ApoA-I priming

**Fig 5:**
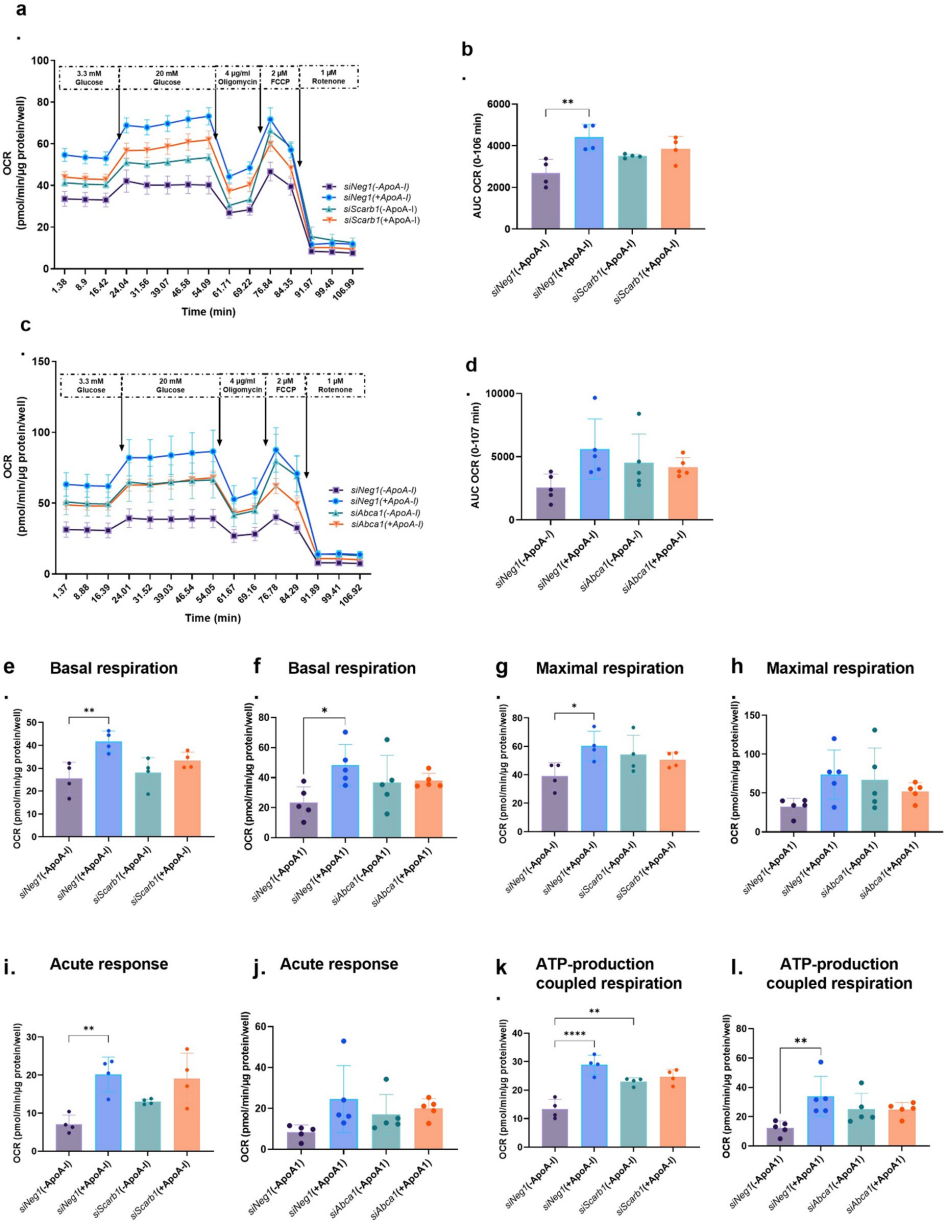
Effects of ApoA1 priming on oxidative phosphorylation (OXPHOS) in siScarb1 (SR-BI) and siAbca1 (ABCA1) silenced INS-1E cells. a & c. OCR (oxygen consumption rate), b & d. AUC for OCR, e & f. basal respiration, g & h. maximal respiration, i & j. acute response and k & l. ATP-production coupled respiration in siScarb1 (SR-BI) or siAbca1 (ABCA1) versus siNeg (Control) in INS-1E silenced cells with or without ApoA-I priming (0.5mg/ml) for 2h (n≥4). Each value represents mean ± SD, except in a and c where it is mean ± SEM; *p<0.05, **p<0.01, ****p<0.0001; unpaired one-way-ANOVA with Bonferroni posthoc test.

After confirming ApoA-I plays a role in mitochondrial metabolism, we determined if silencing SR-BI or ABCA1 influences ApoA-I effects on mitochondrial metabolism (Fig 5 and S6 Fig in S1 File). ApoA-I priming (0.5 mg/ml) in the *siNeg* control cells showed the same effect as seen in Fig 4 with untransfected cells. When SR-BI (Fig 5a and 5b) or ABCA1 (Fig 5c and 5d) are silenced, however, this OCR increase induced by ApoA-I is no longer significant. Similar patterns are seen for basal respiration, maximal respiration, acute response and ATP-production coupled respiration (Fig 5e–5j) with increases occurring upon ApoA-I priming and a lack thereof when either SR-BI or ABCA1 are silenced.

### Mitochondrial pathways are associated with type 2 diabetes genes correlated with SR-BI and ABCA1 in human β-cells

Next, we were interested to find if SR-B1 and ABCA1 negatively associated with genes differentially expressed in human β-cells from T2D donors. Indeed, 73 and 3 genes differentially expressed in β-cells from donors with and without type 2 diabetes were negatively correlated with SR-BI and ABCA1 respectively (Tables 2 & 3). In MitoCarta3.0, four of the 73 SR-BI correlated genes and one of the three ABCA1 correlated genes were associated with mitochondrial pathways (Tables 2 & 3). Further investigation of SR-BI correlated genes by GSEA with WebGestalt also found significant enrichment in passive and metal ion transmembrane transporter activity (FDR ≤ 0.05). This was associated with an additional four of the 73 correlated genes (Tables 2 & 3, S1 Table in S1 File).

**Table 2:** Summary of SR-BI correlation and pathway analysis with β-cell differential expression data from human donors with vs without type 2 diabetes. *Significant pathways identified by Webgestalt.

Gene	r	p-value	FDR	MitoCarta and Webgestalt^a^ Pathway Identification
***DCAF13P3***	-0.881	0.00034	0.03041	
***CPSF2***	-0.862	0.00065	0.03596	
***PSMD1***	-0.860	0.00069	0.03660	
***FIS1***	-0.857	0.00074	0.03744	Mitochondrial dynamics and surveillance, Fission
***RAB3B***	-0.857	0.00075	0.03760	
***SCN3A***	-0.852	0.00087	0.03919	Metal ion transmembrane transporter activity^a^, Passive transmembrane transporter activity*
***ENOSF1***	-0.852	0.00087	0.03928	
***PLEKHA6***	-0.847	0.00101	0.04094	
***APOBEC2***	-0.846	0.00104	0.04132	
***THBS3***	-0.843	0.00111	0.04218	
***PPP1R1C***	-0.827	0.00169	0.04818	
***HARS***	-0.821	0.00195	0.05044	Mitochondrial central dogma, Translation, mt-tRNA synthetases
***PRUNE2***	-0.811	0.00245	0.05452	
***LINC00643***	-0.810	0.00249	0.05478	
***RAB1B***	-0.807	0.00267	0.05614	
***SCRN1***	-0.806	0.00271	0.05641	
***CLDN11***	0.800	0.00314	0.05937	
***CD9***	-0.796	0.00341	0.06110	
***RPN2***	-0.794	0.00355	0.06200	
***UBR1***	-0.791	0.00371	0.06294	
***ALDH18A1***	-0.790	0.00382	0.06360	Metabolism, Amino acid metabolism, Proline metabolism
***NDUFS8***	-0.788	0.00396	0.06439	OXPHOS, OXPHOS subunits, Complex I, CI subunits, Metabolism, Metals and co-factors, Fe-S-containing proteins
***POLR3H***	-0.788	0.00397	0.06444	
***LOC101927151***	-0.787	0.00405	0.06493	
***CDK10***	-0.786	0.00412	0.06530	
***KIAA1324***	-0.786	0.00413	0.06535	
***TP53BP1***	-0.785	0.00423	0.06592	
***SATB1***	-0.782	0.00445	0.06710	
***PUF60***	-0.781	0.00454	0.06765	
***PSMD8***	-0.780	0.00466	0.06830	
***RGS9***	-0.777	0.00486	0.06940	
***CD81***	-0.774	0.00514	0.07085	
***ALOX12*.*AS1***	0.774	0.00518	0.07107	
***MAP7D2***	-0.772	0.00539	0.07208	
***FAM114A1***	-0.771	0.00552	0.07271	
***B9D1***	-0.766	0.00601	0.07509	
***VKORC1***	-0.765	0.00603	0.07520	
***MED22***	-0.759	0.00671	0.07834	
***SPAG1***	-0.757	0.00701	0.07970	
***SEPN1***	-0.755	0.00721	0.08059	
***LDLRAD3***	-0.755	0.00725	0.08076	
***AKR1A1***	-0.754	0.00734	0.08113	
***CAMK2B***	-0.754	0.00739	0.08135	
***ANKRD36B***	-0.753	0.00750	0.08183	
***RABAC1***	-0.752	0.00760	0.08224	
***ACTL6B***	-0.751	0.00767	0.08254	
***LPPR2***	-0.751	0.00769	0.08263	
***ATP5D***	-0.751	0.00774	0.08284	
***ARFIP2***	-0.746	0.00838	0.08544	
***ZBTB20*.*AS1***	-0.746	0.00844	0.08568	
***APOL2***	-0.742	0.00888	0.08743	
***MAGED1***	-0.741	0.00908	0.08825	
***COPZ1***	-0.741	0.00913	0.08841	
***GAS6***	-0.740	0.00918	0.08862	Metal ion transmembrane transporter activity^a^, Passive transmembrane transporter activity*
***KCNK17***	-0.740	0.00927	0.08897	Metal ion transmembrane transporter activity^a^, Passive transmembrane transporter activity*
***KBTBD11***	-0.737	0.00973	0.09070	
***CTU2***	-0.736	0.00976	0.09080	
***HRK***	0.734	0.01017	0.09236	
***ROBO1***	-0.732	0.01048	0.09353	
***GCK***	-0.731	0.01060	0.09392	
***FAM213B*..*1***	-0.730	0.01077	0.09453	
***LGALS3BP***	-0.729	0.01087	0.09490	
***THBD***	-0.728	0.01104	0.09550	
***CELF3***	-0.728	0.01112	0.09578	
***NIPAL4***	-0.727	0.01119	0.09606	Metal ion transmembrane transporter activity*
***C14orf132***	-0.727	0.01124	0.09622	
***HNRNPA2B1***	-0.727	0.01125	0.09627	
***GALNT10***	-0.724	0.01179	0.09813	
***PTP4A3***	-0.723	0.01187	0.09841	
***LDLRAP1***	-0.723	0.01196	0.09872	
***RPLP0P2***	0.723	0.01199	0.09882	
***PEMT***	-0.722	0.01204	0.09898	
***ZGPAT***	-0.722	0.01209	0.09916	

**Table 3:** Summary of ABCA1 correlation and pathway analysis with β-cell differential expression data from human donors with vs without type 2 diabetes.

Gene	r	p-value	FDR	MitoCarta Pathway Identification
***MPC1***	-0.741	0.00901	0.08795	Metabolism, Carbohydrate metabolism, Gluconeogenesis, Pyruvate metabolism, TCA associated, Small molecule transport
***TRIM27***	-0.733	0.01035	0.09305	
***SDHAP2***	-0.726	0.01148	0.09707	

## Discussion

Here, using INS-1E cells we identify and experimentally confirm SR-BI and ABCA1 as the primary receptors for ApoA-I in pancreatic β-cells. Moreover, we demonstrate that ApoA-I-SR-BI and ApoA-I-ABCA1 receptor driven GSIS is significantly mediated by upregulation of β-cell mitochondrial metabolism. Secondary to this, we reveal ***that*** significant loss of either β-cell SR-BI or ABCA1 receptors alone also induces a subtle positive effect on β-cell OXPHOS, and present convincing evidence demonstrating that when SR-BI or ABCA1 signaling are implicated in β-cell GSIS, either mitochondrial and ion channel/ion transporter pathways or solely mitochondrial pathways are involved respectively.

This is the first time an independent screening experiment has been performed to specifically identify the primary receptors for ApoA-I in β-cells. LRC-TriCEPs identifies ligand targets on living cells by capturing stable and transient interactions with high reliability [25, 26]. Here, SR-BI and RMD1 were identified as potential ApoA-I targets in rat INS-1E cells while ABCA1 was considered a borderline enriched target with caution meeting significance but not log2FC criteria for identification (Fig 1 and Table 1). SR-B1, ABCA1 and additional cholesterol transporter ATP-binding cassette transporter 1 (ABCG1) have been functionally investigated as receptors for ApoA-I mediated effects on insulin secretion before in clonal β-cell models, excised islets, or animal models and there is strong evidence supporting SR-BI and ABCA1 involvement [27–32]. RMD1 however is a novel target and has not been investigated as an ApoA-I receptor in any cell line/tissue.

We initially silenced SR-B1, ABCA1 and RMD1 in INS-1E cells (Fig 2) and assessed GSIS in all cell models (Fig 3). Loss of the positive effect of ApoA-I priming on insulin secretion was apparent in SR-B1 and ABCA1 knockdown cells only (Fig 3) demonstrating that ApoA-I binding to SR-BI or ABCA1 but not RMD1 initiates an insulin signaling cascade in β-cells. Our findings here with SR-BI and ABCA1 knockdown are in line with those found before in the mouse MIN-6 β-cell model [30, 32]. For ABCA1, they are also supported by other studies which found insulin secretion impaired in islets excised from transgenic β-cell specific ABCA1 knockout mice. Not surprisingly, these negative effects on *in vivo* insulin secretion have been attributed to accumulation of islet cholesterol and impaired exocytosis of insulin granules [28, 29, 33], and linked to disturbed intracellular Ca^2+^, membrane microdomain organization and Golgi ultrastructure, pro-insulin processing, and insulin granule morphology [28, 33]. Indeed in agreement, cholesterol accumulation in β-cells is reported to unfavorably alter composition of lipid rafts and membrane fluidity leading to decreased glucose transporter levels, impaired GCK activity and ATP generation, changes in spatial organization of L-type voltage gated Ca^2+^ and K_ATP_ channels and ultimately decreased insulin exocytosis [34].

Somewhat relatedly, RMD1 is reported to play a functional role in intracellular microtubule binding [35] and ApoA-I induces trafficking of cholesterol in rat astrocytes via associating microtubules with cytosolic lipid-protein particles [36]. It is thus conceivable RMD1 is also important for intracellular β-cell cholesterol trafficking/homeostasis and insulin granule stability. Nonetheless, considering GSIS was undisturbed by RMD1 knockdown with or without ApoA1 priming in our experiments, we speculate RMD1 does not play an essential role in such processes, at least in healthy β-cells under non-stressed conditions.

Collating previous studies, the current consensus of how ApoA-I triggers insulin release involves ApoA-I interaction with a β-cell surface heterotrimeric G protein Gα_s_ receptor and subsequent cellular internalization, which in turn induces adenylyl cyclase activity leading to increased intracellular cAMP and Ca^2+^, and activation of protein kinase A (PKA), protein kinase C (PKC), janus kinase 2 (JAK2) and/or rho GTPase cell division control protein 42 homolog (Cdc42). These or other unknown mechanisms then promote nuclear exclusion of forkhead box protein O1 (FOXO1), nuclear translocation of PDX1, increased levels of proinsulin processing enzyme protein convertase 1 (PC1/3), mobilization of insulin granules to the cell membrane and ultimately increased insulin output [8, 30, 37].

The classical K_ATP_ dependent pathway involves β-cell conversion of glucose to pyruvate, pyruvate entry into the tricarboxylic acid (TCA) cycle in the mitochondria and metabolism to ATP, an increase in cytosolic ATP and the ATP/ADP ratio, closure of K_ATP_ channels, opening of voltage gated Ca^2+^ channels, Ca^2+^ influx and insulin exocytosis [38, 39]. Importantly, whilst ApoA-I effects on GSIS have also been found dependent on glucose metabolism and ATP-sensitive potassium (K_ATP_) channel activity [37], positive effects of ApoA-I on β-cell mitochondrial activity upstream of the K_ATP_ channel and downstream of glucose metabolism in the K_ATP_ dependent pathway of insulin secretion are not confirmed. Indeed, a recent study found ApoA-I protects β-cells from cholesterol induced mitochondrial damage and restores insulin secretion by colocalizing with mitochondria and reducing oxidative stress. Whilst in this study ApoA-I internalization in INS-1E cells was also linked to cell surface mitochondrial F_1_-ATPase β-Subunit binding, increased cholesterol content, and up- and downregulated expression of genes related to mitochondrial OXPHOS, no effect of ApoA-I on mitochondrial OCR (a proxy for OXPHOS and ATP production) was observed by seahorse analysis [40]. Conversely, our seahorse experiments found mitochondrial OCR/metabolism in cultured INS-1E cells was dose dependently upregulated with ApoA-I priming at concentrations up to 0.5 mg/ml (Fig 4). In follow up investigations, we also found that the ApoA-I OCR effect at 0.5mg/ml was greatly attenuated upon SR-BI or ABCA1 silencing (Fig 5). Together our findings thus demonstrate ApoA-I binding to SR-BI or ABCA1 is a requirement for ApoA-I mediated upregulation of TCA cycle metabolism and mitochondrial OXPHOS in β-cells leading to increased ATP production, and we conclude ApoA-I upregulates K_ATP_ dependent insulin secretion therein via increasing mitochondrial metabolism, cytosolic ATP and the ATP/ADP ratio through SR-BI and ABCA1 receptor binding.

Indeed, ApoA-I has already been positively linked to mitochondrial function in skeletal muscle, cardiac tissue, and primary astrocytes [41–47]. ApoA-I implicated mechanisms include upregulation of OXPHOS/ATP synthesis in skeletal muscle and primary astrocytes [41, 47], as well as stabilization and protection of OXPHOS complexes from oxidative damage, and upregulation of survival signaling pathways to safeguard mitochondrial function in cardiac tissue [42–44]. Of relevance, investigations in macrophages also suggest the latter likely occurs via ABCA1 [45, 46].

As stated, SR-BI or ABCA1 silencing in INS-1E cells attenuated the OCR response to ApoA-I priming in our experiments. OCR was not however completely abolished in either silenced cell model, and similar increases were observed in silenced cells not primed with ApoA-I. Clearly, significant reduction in expression of these ApoA-I receptors also upregulates mitochondrial metabolism independent of ApoA-I binding, albeit to a lesser extent. To delve further and relate our findings for ApoA-I priming with these receptors to human physiology, we explored publicly available β-cell expression data from human donors with (n = 5) and without (n = 6) type 2 diabetes (GSE153855) [19]. We correlated SR-BI and ABCA1 expression with all genes differentially expressed in type 2 diabetes, and then checked significantly correlated genes for pathway associations (Tables 2 and 3).

We found 73 and three genes negatively correlated with SR-BI and ABCA1 respectively. For SR-BI, mitochondrial, and metabolic pathways were associated with FIS1, HARS, ALDH181A1 and NDUFS8 and metal ion transmembrane transporter activity was associated with SCN3A, GAS6, KCNK17 and NIPAL4 correlated genes. For ABCA1, the most significantly correlated gene MPC1 was associated with metabolic mitochondrial pathways including the TCA cycle. The two other ABCA1 correlated genes have been shown to have other functions; TRIM27 was previously reported to facilitate mitophagy [48], and pseudogene SDHAP2 forms part of mitochondrial complex II [49]. SR-BI and ABCA1 associated changes in insulin output are thus linked to mitochondrial and ion channel/exocytotic pathways, and mitochondrial pathways respectively. Overall, we speculate that increased OCR upon SR-BI or ABCA1 knockdown alone is induced by upregulation of mitochondrial and/or ion channel activity suggesting disturbed SR-BI or ABCA1 expression in β-cells instigate a strong compensatory response from other β-cell genes/proteins to maintain metabolic capability. Supporting this, overexpression of cluster of differentiation 36 (CD36), another receptor in the SR-BI family, has previously been shown to impair insulin secretion by downregulating exocytotic processes in INS-1 cells [50].

Individuals heterozygous for ABCA1 mutations have diminished β-cell function [51], and reduced ABCA1 expression has been observed in type 2 diabetes [52]. Furthermore, in addition to the negative effects of ABCA1 loss/cholesterol accumulation on β-cell function reported by others and noted above, excess cholesterol can accumulate in the mitochondrial membrane leading to reduced ETC capacity as well as increased ROS production and mitochondrial stress which in turn gives rise to apoptosis [34]. The positive effect of ABCA1 silencing on mitochondrial pathways/metabolism irrespective of GSIS inhibition observed in our cells therefore suggests they may be suffering from cholesterol accumulation detrimental to β-cell mechanisms/output but not yet serious enough to compromise mitochondrial membrane fluidity and activity. It is indeed possible the cells are attempting to manage high extracellular glucose levels by upregulating intracellular mitochondrial metabolism and thus ATP production in the insulin secretion pathway. Perhaps both ABCA1 and SR-BI have expression thresholds in β-cells for efficient compensation via upregulation of mitochondrial associated genes and pathways. A thorough exploration of the underlying ApoA-I -SR-BI/ABCA1 mechanisms in the β-cell is however required to fully investigate.

Amongst other possible follow up investigations to our study such as assessing vitamin D and E in association with ApoA-I priming for improving insulin secretion to both treat diabetes and prevent diabetes complications [53, 54], a suggested starting point is to silence both SR-BI and ABCA1 simultaneously and examine INS-IE mitochondrial function and insulin secretion to investigate diminished effects and possible compensatory mechanisms. Whilst the main strength of our study is that we have for the first time experimentally proven mitochondrial OXPHOS is integral to ApoA-I-SR-BI and ApoA-I-ABCA1 mediated insulin secretion, a limitation is that the TriCEPs assay captures only sterically accessible glycans [21], and therefore, we cannot exclude that there may be other ApoA-I receptors in β-cells which we have not identified to follow up functionally. Furthermore, we have performed all our functional experiments in INS-IE cells only. Whilst we could not validate our findings in human islets due to lack of availability, we have however instead exploited human data to demonstrate our findings are clinically translatable. This is also a major of strength of our study since validation of ApoA-I findings with regards to β-cell function in human cells and sample data is very limited in the current literature.

## Conclusions

In summary, we present strong evidence that ApoA-I priming potently induces insulin secretion in β-cells via significant upregulation of mitochondrial metabolism. Further to this we claim SR-BI and ABCA1 as the primary receptors by which ApoA-I mediates these actions and confirm associations of SR-BI with mitochondrial and ion channel/ion transporter pathways and of ABCA1 with mitochondrial pathways in β-cells from human donors. Together our findings demonstrate the importance of ApoA-I-SR-BI and ApoA-I-ABCA1 interactions for optimizing β-cell function. Moreover, we have uncovered ApoA-I-SR-BI and ApoA-I-ABCA1 novel interaction pathway targets for improving insulin secretion to prevent and treat diabetes.

## Supporting information

S1 File Containing S1-S6 Figs and S1 Table. (DOCX)
