# Supplementary material for: Apolipoprotein A-I priming via SR-BI and ABCA1 receptor binding upregulates mitochondrial metabolism to promote insulin secretion in INS-1E cells
Source: PLoS One. 2024 Nov 15;19(11):e0311039. doi: 10.1371/journal.pone.0311039 (PMC11567530; doi:10.1371/journal.pone.0311039)
Supplement: S1 File — (DOCX) [file pone.0311039.s001.docx]

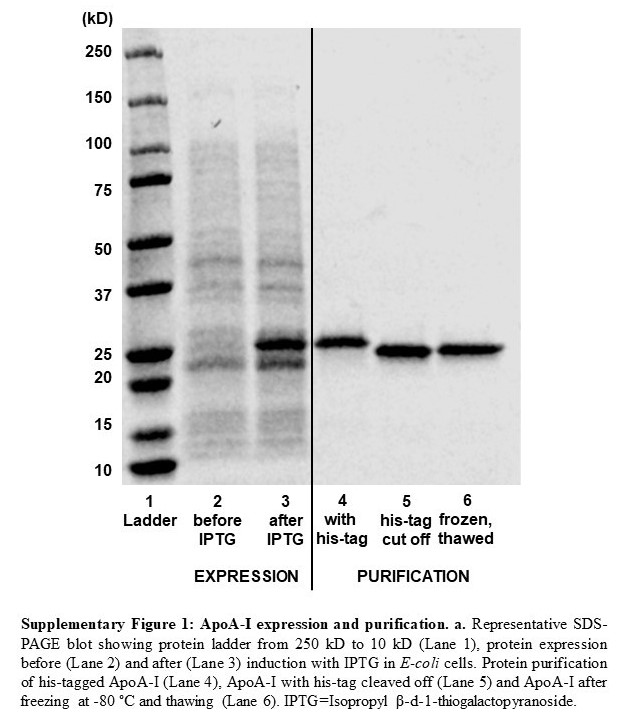


# Supplementary Figure 1: ApoA-I expression and purification. a. Representative SDS-PAGE blot showing protein ladder from 250 kD to 10 kD (Lane 1), protein expression before (Lane 2) and after (Lane 3) induction with IPTG in *E-coli* cells. Protein purification of his-tagged ApoA-I (Lane 4), ApoA-I with his-tag cleaved off (Lane 5) and ApoA-I after freezing at -80 °C and thawing (Lane 6). IPTG*=*Isopropyl β-d-1-thiogalactopyranoside.


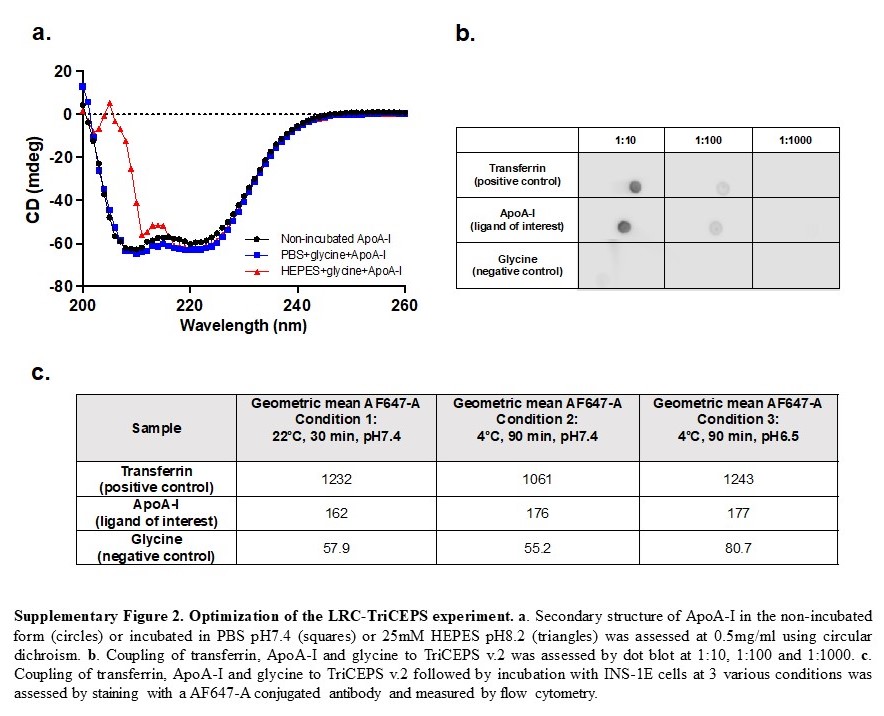


# **Supplementary Figure 2.** Optimization of the LRC-TriCEPS experiment. a. Secondary structure of ApoA-I in the non-incubated form (circles) or incubated in PBS pH7.4 (squares) or 25mM HEPES pH8.2 (triangles) was assessed at 0.5mg/ml using circular dichroism. b. Coupling of transferrin, ApoA-I and glycine to TriCEPS v.2 was assessed by dot blot at 1:10, 1:100 and 1:1000. c. Coupling of transferrin, ApoA-I and glycine to TriCEPS v.2 followed by incubation with INS-1E cells at 3 various conditions was assessed by staining with a AF647-A conjugated antibody and measured by flow cytometry.

# **Supplementary Figure 3.** Protein topology plots. Protein topology plots for a. SCRB1 (SR-BI), b. RMD1 and c. F1LNL3 (ABCA1) illustrate the sequence, topology and annotations for the proteins (data generated using Protter [23], SCRB1: <https://bit.ly/3j1RDAl>, RMD1: <https://bit.ly/2SV5DkS>, F1LNL3: <https://bit.ly/2SPqKoS>).

#


**Supplementary Figure 4:** Western immunoblots in INS-1E silenced cells. Blot 1 is the protein expression assessment for SR-BI in *siNeg* and *siScarb1* cells a. the lane layout, b. SR-BI expression and c. total protein. Blot 2 is the protein expression assessment for ABCA1 in *siNeg* and *siAbca1* cells d. the lane layout, e. ABCA1 expression and f. total protein. n=4 for each.

#
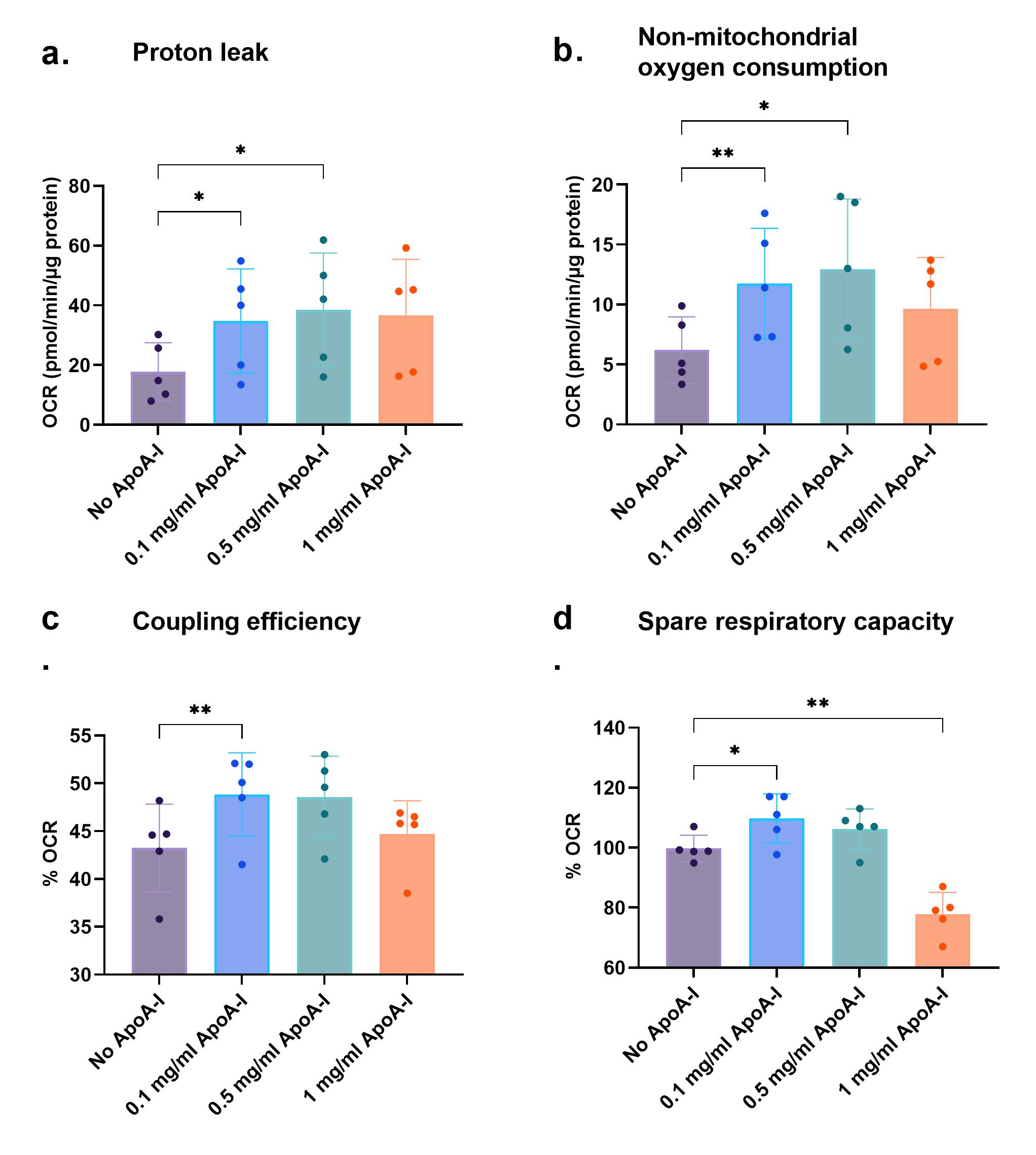
Supplementary Figure 5: Effects of ApoA-I priming on oxidative phosphorylation (OXPHOS) in cultured INS-1E cells. a. proton leak, b. non-mitochondrial oxygen consumption, c. coupling efficiency and d. spare respiratory capacity in INS-1E cells with or without ApoA-I priming (0.1mg/ml, 0.5mg/ml or 1mg/ml) for 2h (n=5). Each value represents mean ± SD; *p<0.05, **p<0.01; paired one-way ANOVA with Dunnett’s posthoc test.

# **
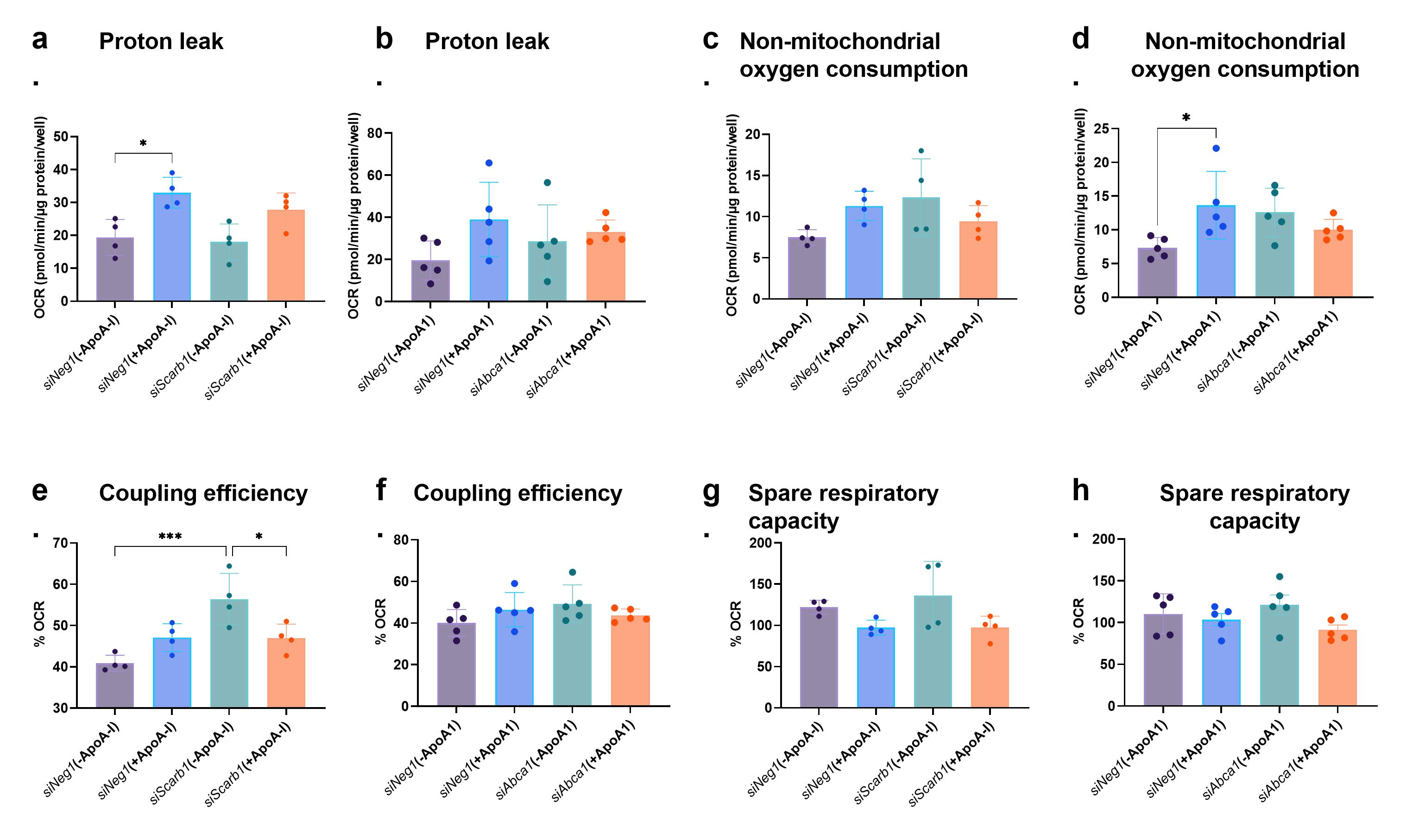
**

# **Supplementary Figure 6:** Effects of ApoA-I priming on oxidative phosphorylation (OXPHOS) in *siScarb1* (SR-BI) and *siAbca1* (ABCA1) silenced INS-1E cells. a & b. proton leak, c & d. non-mitochondrial oxygen consumption, e & f. coupling efficiency and g & h. spare respiratory capacity in *siScarb1* (SR-BI) or *siAbca1* (ABCA1) versus *siNeg* (Control) INS-1E silenced cells with or without ApoA-I priming (0.5mg/ml) for 2h (n≥4). Each value represents mean ± SD; *p<0.05, ***p<0.001; unpaired one-way-ANOVA with Bonferroni posthoc test.

# **Supplementary Table 1.** GSEA results (ontology and molecular function) for the 73 differentially expressed and SR-BI correlated T2D genes in β−cells from human donors.

| **Gene Set** | **Description** | **Size** | **ES** | **NES** | **p-value** | **FDR** |
| --- | --- | --- | --- | --- | --- | --- |
| GO:0046873 | Metal ion transmembrane transporter activity | 4 | 0.90769 | 1.8315 | 0.0030120 | 0.0015244 |
| GO:0022803 | Passive transmembrane transporter activity | 3 | 0.89394 | 1.6246 | 0.0064412 | 0.020198 |
